# Supplementary material for: A Global Screen for Assembly State Changes of the Mitotic Proteome by SEC-SWATH-MS
Source: Cell Syst. 2020 Feb 26;10(2):133–155.e6. doi: 10.1016/j.cels.2020.01.001 (PMC7042714; doi:10.1016/j.cels.2020.01.001)

P63272 | SPT4H_HUMAN | SUPT4H1 SPT4H SUPT4H
Monomer MW [kDa]: 13.193 Monomer expected elution fraction: 58

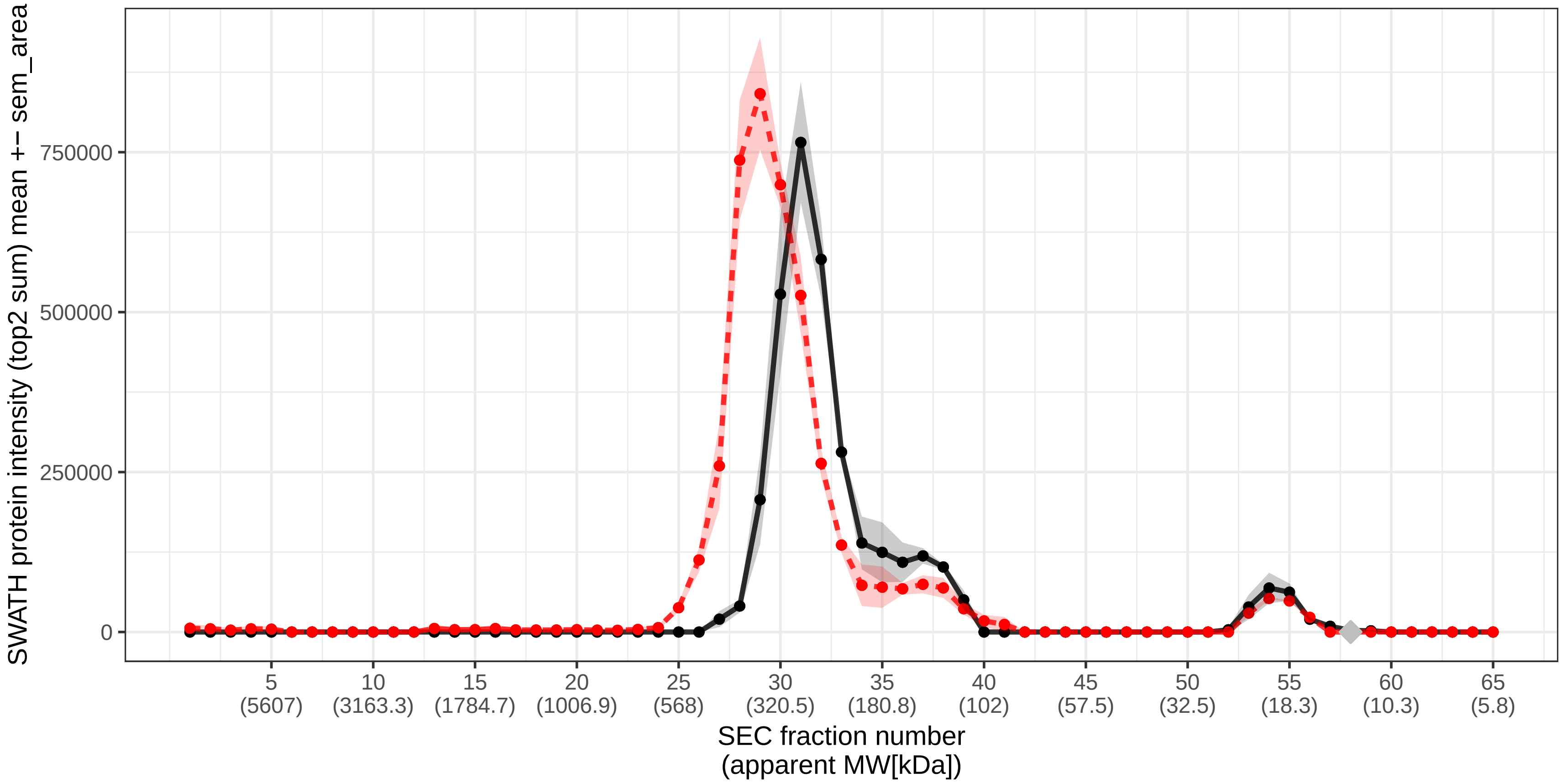

Supplement: Data S1. SEC-SWATH-MS Protein Chromatograms, Related to Figure 1 [file mmc6.zip › SECchrom_P63272_SPT4H_HUMAN_SUPT4H1_SPT4H_SUPT4H.pdf]
